# Lower Physical Performance in Colder Seasons and Colder Houses: Evidence from a Field Study on Older People Living in the Community

**DOI:** 10.3390/ijerph14060651

**Published:** 2017-06-17

**Authors:** Yukie Hayashi, Steven M. Schmidt, Agneta Malmgren Fänge, Tanji Hoshi, Toshiharu Ikaga

**Affiliations:** 1 School of Science for Open and Environmental Systems, Graduate School of Science and Technology, Keio University, Hiyoshi 3 14 1, Kohoku, Yokohama 2238522, Japan; ikaga@sd.keio.ac.jp; 2 Department of Health Sciences, Faculty of Medicine, Lund University, Box 157, Lund 22100, Sweden; steven.schmidt@med.lu.se (S.M.S.); agneta.malmgren_fange@med.lu.se (A.M.F.); 3 Department of Urban Science, Tokyo Metropolitan University, Minamiosawa 1 1, Hachioji, Tokyo 1920397, Japan; star@onyx.dti.ne.jp

**Keywords:** indoor thermal environment, field study, frail, physical strength examination

## Abstract

The aim of this paper was to explore the effect of seasonal temperature differences and cold indoor environment in winter on the physical performance of older people living in the community based on a field study. We recruited 162 home-dwelling older people from a rehabilitation facility in the Osaka prefecture, Japan; physical performance data were available from 98/162 (60.5%). At the same time, for some participants, a questionnaire survey and a measurement of the indoor temperature of individual houses were conducted. The analysis showed that there were seasonal trends in the physical performance of older people and that physical performance was worse in the winter compared with the autumn. Furthermore, people living in colder houses had worse physical performance. The findings indicate that keeping the house warm in the winter can help to maintain physical performance.

## 1. Introduction

Aging is related to declining physical performance leading to functional limitations and disability in the elderly [1,2,3]. Low physical performance is known as a risk factor of falls [4,5], avoidance of activities [6], and sarcopenias [7], which are related to frailty. There is evidence from experimental studies that cold exposure may reduce physical performance [8,9,10,11,12]. It has been found that the working capacity of muscle [8,9], maximal force production [10], and the time needed to attain maximal force [11] are all degraded by cooling. Furthermore, Oksa et al. [12] found that even mild cooling has a negative effect on physical performance.

Older people may have a higher risk of failure to maintain core temperature during exposure to a cold environment because of a higher level of skin thermal conduction [13] and reduced reflex vasoconstriction [14]. Based on an experimental trial, Lindemann et al. [15] showed that the physical performance of older women was worse in 15 °C room temperature compared with 25 °C room temperature. 

Experimental trials on the effect of temperature on performance usually only have a few hours of exposure, in which the participant’s environment and behavior, in particular their activity and clothing, are rigidly controlled and usually kept constant [14]. Little is known about long-term exposure to lower household temperatures. There is some evidence of an association between low outdoor temperature and fall-related hip fracture hospitalizations in older people [16] but not about indoor temperature. Evidence from indoor temperature has mainly been limited to the association between colder indoor temperatures with a higher blood pressure [17,18].

In order to prevent cold-induced adverse effects among older people, it is important to recognize cold-related reduction of physical performance and to identify the individual factors that increase or decrease the risk of frailty posed by cold ambient temperatures. Furthermore, since older people spend most of their time in their home environment [19], not only outdoor temperature in the colder seasons but also indoor temperature in the winter may affect their physical performance. Houses in Japan typically have cold indoor temperatures in the winter. This is mainly due to low insulation and partial heating systems in the form of an electric foot warmer [20]. However, evidence remains limited on the determinants of vulnerability particularly in relation to socio-economic factors including fuel poverty, and the role of thermally inefficient housing in Japan. The Japanese policy has a clear preference for non-residential building, and thermal regulations are limited to large developments only (over 300 m^2^) and exclude most of the residential sector. As the implementation of environmental policies is voluntary, and the insulation levels low, the low heating demand per household seems to be due to a different behavioral culture [20].

The aim of this study was to investigate the effect of seasonal temperature differences and cold indoor environments in the winter on the physical performance of older people living in the community. We hypothesized that there are seasonal trends in the physical performance of older people, with worse physical performance in the winter compared to intermediate seasons such as the autumn. The secondary hypothesis was that people living in colder houses would have a greater reduction in physical performance compared to those in warmer houses.

## 2. Materials and Methods 

### 2.1. Participants

In December of 2014 and 2015, we recruited 162 home-dwelling elderly volunteers through a rehabilitation facility in the Osaka prefecture, Japan. All of the participants used this facility once or twice a week for physical rehabilitation. Participants’ characteristics, including age, gender, body mass index, and economic satisfactions were obtained using a standardized questionnaire. Characteristics of their housing, including building age, living period, and the number of window glass panes, were also obtained in this questionnaire. A subsample agreed to allow the research team to assess indoor temperature of their homes, and these complete indoor temperature measurements taken over a period of 2 weeks in December were employed in the analysis of indoor temperature (*n* = 36). The study protocol was approved by the Keio University Ethics Review Board on 4 August 2014 (26-11).

Exclusion criteria for the analysis of seasonal differences were as follows: (i) a lack of physical performance data; (ii) a lack of physical performance data assessed in the winter (December–February); (iii) a lack of physical performance data assessed in the autumn (September–November). Because the study was conducted in December, some participants using the rehabilitation facility for less than a year had not completed the physical performance assessment, but it was planned for January or February; therefore, they were excluded from the analysis. Baseline data on 98 participants were included in the analysis of seasonal differences in physical performance, and temperature data from 36 participants were included in the analysis of indoor temperature. The 36 participants in the analysis of indoor temperature are a subsample of the 98 participants in the analysis of seasonal differences in physical performance.

### 2.2. Measurement of Physical Performance

All the data on physical performance were collected from records in the rehabilitation facility. The primary assessment was performed when people began using the facility and was then repeated every 3 months. Assessed items were grip strength (kg), static postural and balance control assessed by single-leg standing time (s), and balance and gait function assessed by the Timed Up & Go (TUG) test (s). Grip strengths were measured with dynamometer TKK 5001 (Takei Scientific Instruments Co., Nigata, Japan) in the seated position. The best performance of two trials was selected for each side. Single-leg standings were performed with eyes open and arms on the hips without assistance on one leg and were timed in seconds from the time one foot was flexed off the floor to the time when either it touched the ground or the standing leg or an arm left the hips. Single-leg standing was assessed in both legs. Measurement of grip strength and single-leg standing were based on a deployment plan for a physical performance test established by the Ministry of Education, Culture, Sports, Science and Technology (MEXT) in Japan [21]. TUG tests were introduced in 1991 by Podsiadlo and Richardson [22] as a modification of the Get-Up and Go Test of Mathias et al. [23]. TUG tests were timed in seconds from the time to rise from the chair, walk 3 m, turn around a corner, walk back to the chair, and sit down. In TUG tests, both right turns and left turns around the corner were assessed.

### 2.3. Measurement of Indoor Temperature

Temperatures were measured at 10 min intervals 1.1 m above the floor in living rooms, bedrooms, and dressing rooms for approximately 2 weeks from 8 to 26 December 2014 or 2015. Temperatures in living rooms and bedrooms were measured by using data loggers RTR-503 (T&D Corporation, Nagano, Japan) with an accuracy of ±0.3 °C from 0 to 55 °C and a 0.1 °C resolution, and temperatures in dressing rooms were measured using data loggers RTR-501 (T&D Corporation) with an accuracy of ±0.5 °C from −40 to 80 °C and a 0.1 °C resolution. We had no control and did not collect information about the types of clothing people wore when they were at home.

### 2.4. Statistical Analysis

For continuous variables with a normal distribution, mean ± standard deviation (SD) was reported. As static postural and balance control had a skewed distribution, the logarithm value was used in the analysis. Physical performance assessments from the rehabilitation facility in autumn (from September to November) and winter (from December to February) were compared by using a paired *t*-test. When we assessed the changing rate of physical performance between autumn and winter among individual attributes, a simple linear regression analysis was used and partial regression coefficients were reported. Participants who measured indoor temperature of their houses were classified into the warm group and the cold group based on the room temperature recommended in Cold Weather Plan for England 2015 [24]. The recommendation is as follows: “Heating homes to at least 18 °C (65 °F) in winter poses minimal risk to the health of a sedentary person, wearing suitable clothing.” Because of cold housing in Japan due to low thermal insulation, the mean temperature rather than the minimum temperature of living room was used for classification. Using the recommended indoor temperature from the Cold Weather Plan for England 2015, 8 participants were classified into the warm group, and 28 participants were classified into the cold group based on measured indoor temperature during winter. The characteristics of each group were compared using a chi-square test for categorical variables and a Student’s *t*-test for continuous variables. Physical performances in autumn and winter were compared for both groups by using a paired *t*-test in each group. All *p*-values were two-sided, and *p* < 0.05 was considered statistically significant. All statistical analyses were performed with SPSS ver. 22.0 software (IBM, Armonk, NY, USA).

## 3. Results

### 3.1. Baseline Analysis

Of the 98 participants in the baseline analysis (mean age ± SD: 79.4 ± 7.69 years), 54 (55.10%) were women. Most houses had only one glass pane in the windows, and the mean temperature was less than 18 °C in all three rooms. For more details, see Table 1.

### 3.2. Seasonal Differences of Physical Performance

Performance on grip strength and logarithm single-leg standing were worse in the winter compared with the autumn, while performance of balance and function were not significantly different between autumn and winter (Table 2). The differences on grip strength ranged from 3 to 7%.

The changing rate of physical performance between autumn and winter had no significant association with individual attributes in the simple linear regression analysis (Table 3).

### 3.3. Influence from Cold Housing on Seasonal Differences of Physical Performance

**Figure 1:**
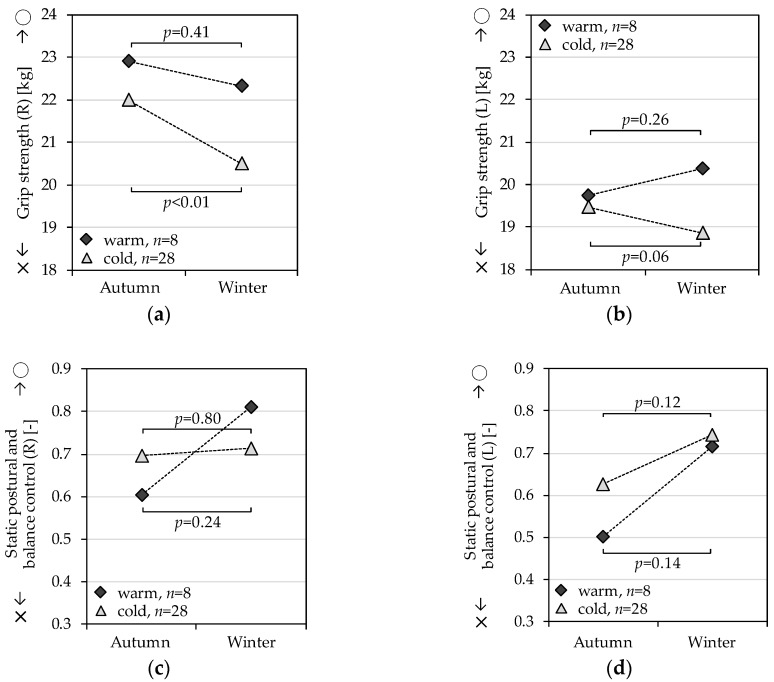
Paired *t*-test of physical performance assessed in autumn and winter grouped by living room temperature. (**a**) Grip strength, R; (**b**) Grip strength, L; (**c**) Static postural and balance control, R; (**d**) Static postural and balance control, L.

The cold group had significantly weaker grip strength in the right hand, while the warm group did not have significant differences between autumn and winter in the right hand (Figure 1a). Grip strength in the left hand showed a divergent pattern between the groups. The cold group had significantly weaker grip strength in the left hand, while the warm group had stronger grip strength in the left hand, but the differences were not significant (Figure 1b). Logarithmic single-leg standing on right and left legs was longer in the winter in both groups, and the warm group had the greatest difference between seasons in terms of the single-leg standing time (Figure 1c,d). Although the clinical relevance of the amount of differences between seasons in the physical performance in our study may be low, all results point in the same direction. However, this difference was opposite of that of the population trend shown in Table 2. 

Moreover, there were no significant difference in characteristics between people living in cold and warm houses (Table 4). 

## 4. Discussion

Our results demonstrated seasonal trends and differences in physical performances between residents in cold or warm houses. The results of grip strength and single-leg standing tests confirm our hypothesis that physical performance is worse in the winter compared to the autumn, which is an intermediate season in Japan. Furthermore, it seems quite probable that people living in colder houses had even worse grip strength. These results suggested that keeping warm during the winter may prevent a decline of physical performance. People in cold houses might only be using partial heating systems in the form of an electric foot warmer, which can narrow their activity scope inside houses, and may easily deteriorate their physical performance. 

On the other hand, balance and gait function showed no relationship with seasonal variation. In a recent review, Schoene [25] found that in high-functioning older adults, TUG tests had a ceiling effect, making it difficult to identify or detect differences in this cohort. Since most of the participants in this study using the facility for physical rehabilitation had various kinds of disability, some participants had a ceiling effect and some did not, which made it difficult for us to detect the effect of thermal environment. Moreover, the single-leg standing test may have difficulty identifying non-seasonal factors since it had a skewed distribution in changing rate between autumn and winter. This trend matches other studies [26] and, furthermore, follow-up data reviewed by Howe et al. [27] including 173 participants in 3 studies [28,29,30] reported that the effectiveness of exercise on single-leg standing time showed no statistically significant difference between these and control groups at six months.

Regardless, this study provides some evidence for the clinical importance of maintaining a warm indoor environment to improve/maintain grip strength. Prolonged exposure to cold indoor temperatures may result in clinically relevant deceases in grip strength, especially in frail older people, which is associated with increased risk for future disability [31,32,33,34], hospitalization [35], and all-cause mortality [32,34,35]. A warm indoor environment can be a preventive measure against these negative outcomes.

### Limitations

The effects of temperature on physical performance may have been underestimated because the value of physical performance used in this study was assessed in a rehabilitation facility rather than in participants’ own houses. Indoor temperature in the rehabilitation facility was not actually measured, but we assumed that it was kept generally comfortably warm. Suggesting a direct association between temperature and physical performance, this fact was not considered in the analyses. This practice was confirmed by comparable results during different seasons. Ramos et al. [36] showed that, in mild climate countries, indoor temperature has great variability in the winter but not in the autumn. If we could assess physical performances in their houses, the exact environments to which participants are exposed, the difference in physical performance between warm houses and cold houses may have been found to be significantly larger. This can also be said for the seasonal differences in physical performance, with even poorer performances in the winter compared to the autumn. Moreover, if we could measure indoor temperature in the autumn as well, it may have been indicated that autumn had a warmer indoor temperature than did the winter. This would have helped us to strengthen the evidence we acquired regarding the seasonal change of indoor temperature, but unfortunately we could not secure thermometers in this case for additional data collection. 

The results of this study cannot be generalized, as this was a convenience sample, although they are in line with other studies based on experiments with older adults [15]. Although residents in colder houses had the lowest grip strength, we must note that the sample size for this result was limited to 8 and 28 in the warm and cold groups, respectively. Because the average insulation is low in Japan, we could not find a larger warm sample. With this sample size, we were not able to evaluate other relevant factors that could influence physical performance, such as sex, age, chronic illness, and obesity, all of which are related to frailty [37]. Although we were not able to conduct a multivariate analysis including these factors due to our small sample size, we did confirm that there were no differences in individual attributes between people living in cold houses and warm houses. Then, by comparing physical performances in each individual participant using paired *t*-tests, we attempted to exclude an effect of an individual difference. As a potential association between physical performance and indoor temperature was found in this study, despite the small sample size and given the other limitations discussed, this study may serve as a feasibility study, which could be used to guide the design and implementation of a larger cohort study in the future. In addition, to better document chronic conditions and disease progression during the study period, a standardized research questionnaire would provide better data than relying on available clinical data.

## 5. Conclusions 

Our study focused on the physical performance of frail elderly people living in cold houses in the winter. The results showed that grip strength was weaker in colder seasons and colder houses. This knowledge can serve as a method to prevent the decline of physical force by improving housing environments. As maintaining grip strength is regarded as a part of care prevention, this study showed an incentive for administration and individual consumers to make an investment in heating and insulation of houses.

## Figures and Tables

**Table 1:** Basic characteristics of participants and their housing environments.

Variable	Participants from Physical Performance Assessment (*n* = 98)		Participants with Indoor Temperature Measurement (*n* = 36)	
Age, (years), mean (SD)	79.43	(7.69)	81.38	(5.81)
BMI, (kg/m^2^), mean (SD)	22.73	(3.13)	22.85	(3.12)
Female, *n* (%)	54	(55.10)	19	(52.78)
Economic satisfaction, *n* (%)				
Very satisfied	9	(9.18)	6	(16.67)
Somewhat satisfied	67	(68.37)	20	(55.56)
Not very satisfied	14	(15.29)	7	(19.44)
Not satisfied at all	7	(7.14)	3	(8.33)
No answer	1	(1.02)	0	(0.0)
Building age, *n* (%)				
0–10 years	11	(11.22)	5	(13.89)
11–20 years	9	(9.18)	2	(5.56)
21–30 years	24	(24.49)	7	(19.44)
31–40 years	18	(18.37)	9	(25.00)
More than 41 years	35	(35.71)	13	(36.11)
Window glass panes, *n* (%)				
One glass pane	71	(72.45)	26	(72.22)
Two glass panes	27	(27.55)	10	(27.78)
Room temperature, (°C) mean (SD)				
Living room			16.76	(1.64)
Bedroom			15.46	(1.83)
Dressing room			14.59	(1.91)

**Table 2:** Paired *t*-test of physical performance assessed in autumn and winter, mean (SD).

Variable	Autumn		Winter		*p*-Value
Grip strength, (kg), R, *n* = 98	21.47	(7.33)	20.05	(7.25)	<0.0001
Grip strength, (kg), L, *n* = 98	19.71	(6.84)	19.09	(7.12)	0.009
Static postural and balance control, (-), R, *n* = 94	0.71	(0.41)	0.61	(0.50)	0.018
Static postural and balance control, (-), L, *n* = 93	0.68	(0.46)	0.59	(0.45)	0.020
Balance and gait function, (sec), R, *n* = 98	12.57	(8.77)	12.61	(7.39)	0.938
Balance and gait function, (sec), L, *n* = 98	12.79	(10.30)	12.58	(7.32)	0.743

**Table 3:** Beta coefficient of physical performance and individual attributes.

Variable	Age (Years)	BMI (kg/m^2^)	Sex	Economic Satisfaction
Grip strength, (kg), R, *n* = 98	0.00	0.00	−0.02	0.01
Grip strength, (kg), L, *n* = 98	0.00	0.00	0.00	−0.02
Static postural and balance control, (-), R, *n* = 94	0.00	−0.01	−0.40	−0.26
Static postural and balance control, (-), L, *n* = 93	−0.01	0.00	0.30	0.02
All variables were not significant.				

**Table 4:** Characteristics of cold and warm groups.

Variable	Cold		Warm		*p*-Value
Sex, *n* (%)					
Male	12	(42.85)	5	(62.50)	0.434
Female	16	(57.14)	3	(37.50)	
Age, (years), mean (SD)	82.08	5.78	79.13	6.10	0.221
BMI, (kg/m^2^), mean (SD)	22.79	3.14	23.05	3.46	0.846
Economic satisfaction, *n* (%)					
Very satisfied	0	(0.00)	0	(0.00)	0.546
Somewhat satisfied	12	(42.85)	5	(62.50)	
Not very satisfied	13	(46.43)	2	(25.00)	
Not satisfied at all	3	(10.71)	1	(12.50)	
